# Therapeutic Nursing Education in Promoting Self-Management of Adolescents with Type 1 Diabetes Mellitus: Integrative Literature Review

**DOI:** 10.3390/nursrep13010043

**Published:** 2023-03-16

**Authors:** Cláudia Pereira, Marta Catarino, Ana Clara Nunes

**Affiliations:** 1 Continuous Care Unit Senhora de Guadalupe, 7830-361 Serpa, Portugal; 2 Health Department, Polytechnic Institute of Beja, 7800-111 Beja, Portugal; 3 Institute of Health Sciences (ICS), Universidade Católica Portuguesa, 1649-023 Lisboa, Portugal; 4 Center for Interdisciplinary Research in Health (CIIS), Institute of Health Sciences (ICS), Universidade Católica Portuguesa, 1649-023 Lisboa, Portugal

**Keywords:** adolescent, Diabetes Mellitus Type 1, nursing, self-management, therapeutic education

## Abstract

Diabetes Mellitus Type I (DM1) is an autoimmune disease, characterized by the total destruction of the beta (β) cells of the islets of Langerhans in the pancreas. This disease can strike people at any age, but it usually develops in children or young adults. Because of the high prevalence of DM1 in the young population, as well as all the difficulties in effective self-management in this population, with very specific characteristics, it is essential to develop therapeutic education interventions, with the aim of acquiring self-management skills. Thus, the main objective of this study is to identify the benefits of therapeutic nursing education interventions in promoting self-management of adolescents with DM1. For this, an Integrative Literature Review was carried out, using the EBSCOhost, PubMed, Scopus and Web of Science platforms. Six articles were eligible. In terms of results, benefits were identified in the health of adolescents, with the use of therapeutic education interventions by nurses, including the control of capillary glycemia, better acceptance of the pathology, improvement in body mass index, increased adherence to the therapeutic regime, a reduction in hospitalizations and complications, contribution to bio-psycho-social well-being and improvement quality of life.

## 1. Introduction

The etiology of Diabetes Mellitus Type I (DM1) can be attributed to a high diversity of pathological and etiological mechanisms. It is an autoimmune disease that arises when an individual has some genetic predisposition with exposure to some precipitating event, for example, bacteria, a viral infection or a chemical agent [1].

DM1 is a chronic disease, making absolute insulin therapy essential to guarantee the survival of the individual [2,3].

Diabetes Mellitus (DM) can be manifested through hyperglycemia that is evidenced by the following signs and symptoms: polydipsia, polyphagia, polyuria, glycosuria, sudden weight loss (despite polyphagia), tiredness, lethargy, blurred vision, dizziness, wounds that heal more slowly and recurrent infections. These manifestations must be identified as early as possible to avoid complications [1,4].

This pathology is among the most common in pediatrics, being recognized as a serious public health problem, since it presents a high prevalence, incidence, morbidity and mortality. It is expanding, with a prevalence rate increasing by approximately 3% per year in children and adolescents and 5% per year in preschool children [5].

This disease is among the most prevalent diseases in childhood and, in particular, in adolescence, with major repercussions on the quality of life of these individuals and, therefore, of their families [6]. Like any chronic disease, it requires the development of behaviors, skills and knowledge, through the necessary and adjusted change in lifestyle and training in disease management in order to avoid the appearance of complications in the short and long term.

In addition, adolescence is defined as a biological process, with the promotion of cognitive development and, consequently, the structuring of the personality [7], and living adolescence with DM1 is a complex process, since this disease requires, for part of the individual, the execution of important tasks for the development of autonomy and independence. This phase of life is characterized by a period of great change, which requires an increased commitment in matters of adaptation to the chronic disease, requiring a differentiated intervention by the health teams [8,9].

In this sense, it is essential to develop strategies in therapeutic education in nursing, to promote self-management of adolescents with DM1, as this process of adaptation to the chronic disease can be among the greatest challenges in the lives of these adolescents [8,10]. Nurses, as first-line health professionals, can develop a privileged work in the self-care management of adolescents with DM1, particularly specialists in Child and Youth Health Nursing [11].

Therapeutic education and monitoring of adolescents with DM1, parents and/or informal caregivers are the key to successful management of the disease. Educational interventions should contemplate a partnership between nurse-adolescent-parents and/or informal caregivers, enabling the sharing of knowledge, so that they accept their new health condition, improve their adaptive abilities in solving problems, as well as its potential, providing an improvement in skills for self-management of the disease [5].

There is evidence in the literature of the benefits of acquiring these skills in the daily life of this population, such as increased satisfaction for both caregivers and those being cared for; improved well-being, health, and quality of life; and reduced personal/family, social, labor, and economic costs [12].

It is important that therapeutic education actions can be carried out in a group (whenever possible), promoting acceptance and consequently the adaptation of these users to this new pathology. The meeting with peers, with the same difficulties, encouraging the sharing of experiences and the development of behaviors and skills that improve their well-being, contribute to the autonomy, management and compensation of DM1 [5]. It is therefore vital that adolescents recognize themselves by (re)constructing their ideas and that they can (re)formulate all their attitudes to deal with their illness.

This literature review enables integrating the literature found on therapeutic nursing education by identifying several studies on the subject. It aims to identify the benefits of therapeutic nursing education interventions in promoting self-management in adolescents with DM1. 

## 2. Materials and Methods

The methodological alignment for carrying out the Integrative Literature Review (ILR) included the following phases: definition of the research question; determination of inclusion and exclusion criteria; definition of descriptors according to the Health Sciences Descriptors; selection of articles based on previously outlined criteria and with the objectives and nature of this study and, finally, analysis, presentation and discussion of results. 

This review was registered in the Open Science Framework (OSF) platform with the DOI 10.17605/OSF.IO/G3SXM.

### 2.1. Research Question

The definition of the research question was elaborated through the PICOD methodological application, where “P” refers to Population—adolescents with DM1; “I” refers to Intervention—therapeutic education interventions in nursing to promote self-management of chronic illness; “C” refers to Comparison (not applicable); “O” refers to Outcome—promotion of DM1 self-management; “D” refers to Design: ILR.

As a result, the research question that guided the present study emerged: “What are the benefits of therapeutic nursing education interventions in promoting self-management in adolescents with Type 1 Diabetes Mellitus?”.

### 2.2. Inclusion and Exclusion Criteria

The defined inclusion criteria include studies with a target population that consists of adolescents with DM1. We followed an adolescent according to the World Health Organization—a person between 10 and 19 years of age; 10 to 14 years of age is considered the pre-adolescent stage, and 15 to 19 years is considered the adolescent stage [13]. The intervention (under analysis in the scientific articles to be included) refers to interventions of therapeutic education in nursing in the promotion of self-management of chronic illness. The outcomes we seek are related to the benefits of interventions. In terms of time horizon, we only included articles published in the last five years (December 2017 to December 2022). Articles in Portuguese, English and Spanish were included, with access to the full text.

Exclusion criteria were all articles that did not meet the criteria and those that were duplicated in the databases where the research was carried out.

### 2.3. Search Strategy

The search for articles was carried out using the EBSCOhost platform (through which we access the specialized databases—CINAHL Complete and multidisciplinary Scopus), the PubMed platform (with access to the specialized Medline database) and the platform Web of Science.

The following descriptors were selected and used: Mesh—“education”, nursing”, “self-management”, “Adolescent”, “Child”, “type 1 diabetes mellitus”, and “type 1 diabetes mellitus”; alternative terms—“therapeutic education”, “nurse”, “nurses”, “self management”, “management self”, “Youth”, “Young people”, “Scholar”, and “diabetes type 1”; truncations—“nurs*”, “Child*”, “Adolescen*”, “Teen*”, “Paediatric*”, “Pediatric*”, and “nursing*”.

The Boolean operators used to combine the various descriptors were: “AND” and “OR”.

To facilitate the understanding of the use of the various descriptors, as well as their combination with the Boolean operators, Table A1 (Appendix A) is presented.

The initial search resulted in 108 articles, of which 13 studies were from CINAHL Complete, 37 studies from PubMed, 17 studies from Scopus and 41 studies from the Web of Science.

**Figure 1:**
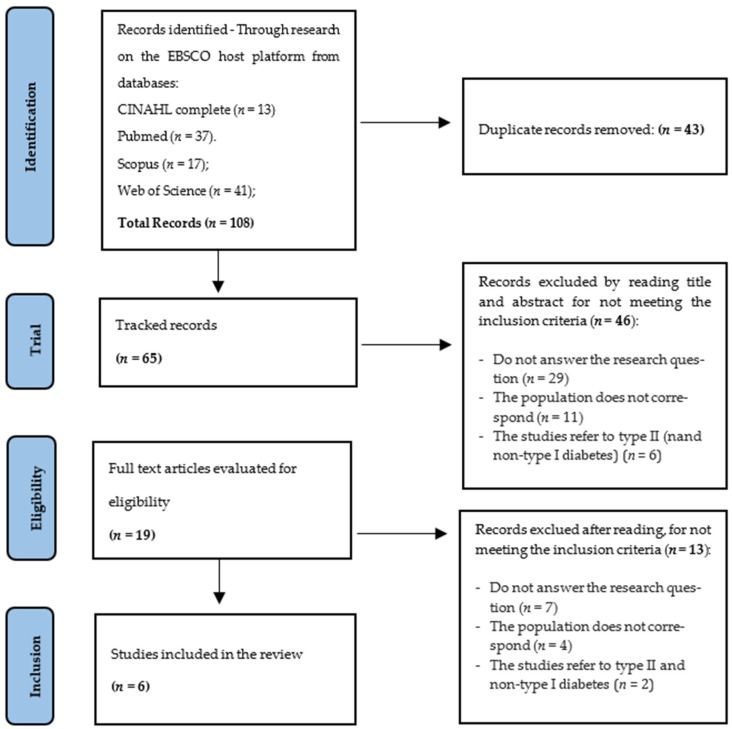
Flowchart based on the PRISMA model.

Subsequently, duplicates were removed using the Endnote software, resulting in 65 articles. Next, the selection of articles for the ILR was carried out by two researchers, independently, and took place in two stages. The first referred to reading the title and abstract of the articles, resulting in 19 articles; in the second stage, we read the full text, making six (6) articles eligible for the ILR. The PRISMA flowchart (Figure 1) outlines and systematizes the strategy pursued to obtain the articles included in this study.

To carry out the evaluation of the methodological quality of the selected articles, as well as the levels of evidence, the contributions developed by the Joanna Briggs Institute [14] were used.

## 3. Results

After the implementation of the PRISMA model, the six articles included in the review were analyzed, which met the inclusion criteria: adolescents (ages between 9 and 18 years old) with DM1, as well as studies carried out in these schools of adolescents, in order to identify interventions to contribute to the therapeutic education of this population and therefore to their self-management of this pathology.

In this way, we present the following Table 1 with the results found.

## 4. Discussion

As an organizing axis, for the analysis and discussion of the benefits resulting from therapeutic education interventions in nursing in promoting self-management of adolescents with DM1, the descriptive statements of the Standards of Quality of Care for Nursing [19].

Currently, guidelines in health advocate the responsibility of individuals with chronic pathologies for self-care, emphasizing the role of health professionals, in particular nurses, in training and therefore in education for acquisition of skills in self-management of health/disease processes [20].

The literature has more recently presented therapeutic education programs to contribute to self-management of chronic conditions, in a holistic way, involving not only treatments and therapy, but also the social and psycho-affective needs of individuals and families [9,21].

About self-management of chronic disease, particularly in DM1, the benefits that may arise for adolescents from therapeutic education interventions on the part of nurses are: a decrease in hospitalizations and future complications, an increase in adherence to the therapeutic regime, an improvement in quality of life and a contribution to the biopsychosocial well-being [9].

In two studies [6,22], it was possible to identify that the interventions carried out by nurses, through motivational interviewing, contributed to improvement in DM1 self-management by adolescents, enabling more detailed care directed to individual needs. In one of these studies [6], two face-to-face sessions lasting approximately 30 min and four telephone contacts resulted in lower HbA1c values, also contributing to changes in the behavior of these adolescents, preparing them for the changes in care as young adults, thus promoting DM1 self-management.

Additionally, in another study [14], the results showed that therapeutic education interventions in nursing, the motivational interview with adolescents with DM1, promoted self-management of the disease. As benefits, they present a decrease in the fear of hypoglycemia, better coexistence with the pathology, more motivation and even an improvement in body mass index.

Colaizzi’s data analysis method [16] enabled us to understand the lived experiences of adolescents in relation to DM1. This study also highlighted parents’ approach to caring for adolescents with DM1, revealing that this approach was not always well accepted by parents, since there are divergences in some points related to the control of DM1, in the desired value in the control capillary blood glucose. In the same study, it emerged that the role of the nurse regarding the encouragement and participation of parents in self-control of DM1 by adolescents is fundamental so that they can achieve their independence, considering the needs presented in the face of DM1 [16].

Regarding the various studies analyzed, five of the six selected articles mentioned motivational interviewing as a therapeutic education intervention in nursing, with benefits in promoting self-management of adolescents with DM1.

By linking the various results, it is possible to state that this intervention enables responding to the descriptive statements of the Quality Standards of Nursing Care, namely customer satisfaction, health promotion, prevention of complications and well-being and self-care [13].

It is also important to highlight that the interventions carried out by the nurse can be carried out at different levels—hospital, primary health care or at school.

The study developed by Bico and Kim [15] is an indicator of this versatility of contexts. In this study, it was evident that the role of nurses in the school context contributes to the development of skills, self-management and self-control of DM1 by adolescents. This follow-up at the school level enables networking with specialists and colleagues, encouraging communication between adolescents with the same pathology, as well as with health professionals or significant others about possible fears or doubts about DM1.

It was also possible to verify, in the study developed by Banca et al. [17], that nurses can carry out interventions outside of the hospital and/or school context. The study was carried out in a summer camp in Brazil using a “therapeutic toy”. During the camp, group sessions were developed with adolescents, where they would have to create and describe drawings about DM1, so they were able to discuss different opinions in a group. These sessions enabled expanding these adolescents’ knowledge about the pathophysiology of DM1, as well as clarifying doubts with the nurses.

Currently, due to the various changes resulting from COVID-19, in the treatment of DM1 in the pediatric population, there is a need to change some education methods. That is, some treatments can be communicated through online platforms or e-learning, which can eliminate some gaps and adapt therapeutic education, promoting DM1 self-care.

We concluded by emphasizing the need to carry out more studies into further approaches to reduce stress, anxiety and fear of changes that may arise unexpectedly among this population, avoiding travel to health institutions, which in many situations entails consequences for the family (for example financially).

## 5. Limitation Heading

This RIL has some limitations, namely that this approach may not be repeatable in different cultures, who may present habits or provision of health care that are different from the Portuguese reality. Additionally, there are studies that are still ongoing that have not been considered. Although there are studies that describe the benefits of therapeutic education interventions, few studies describing these interventions were found. Since adolescence is a pivotal stage of development, with the transition into adulthood, it is essential to invest in studies of this type, identifying interventions that contribute to the promotion of self-management, in this population.

This study, being an integrative review, combined data from theoretical and empirical literature, which may give it a more subjective character.

## 6. Conclusions

DM1 is a chronic disease with a significant impact on the lives of adolescents and their families. Therapeutic education enables adolescents to be trained in disease management, adapting useful methods, contributing to the prevention of complications inherent to the disease and contributing to improvement in quality of life.

The realization of this ILR enabled identifying the benefits associated with interventions of therapeutic nursing education in promoting self-management of adolescents with DM1.

Scientific evidence highlights motivational interviewing as an intervention that promotes DM1 self-management, which enables adolescents to better manage this pathology, a positive perception of their quality of life, and their relationships with their parents/relatives and with their peers.

Regarding therapeutic education, the results obtained demonstrate that this becomes beneficial for self-management of chronic illness and should be a fundamental area in policies of health, since, in situations where the disease is being adequately controlled, health expenses become high, both for families and health institutions.

With the growing development of knowledge about child health as well as current technological advances, we are witnessing an increase in the average life expectancy and the emergence of resources that contribute to adolescents’ autonomy, which was less likely a few decades ago [9].

The acquisition of self-management skills enables preventing complications in adolescents, promoting the proper functioning of their bodies and, therefore, maintaining their health and preventing sequelae at the micro and macrovascular level.

In summary, the perception of adolescents and their families about the importance of self-management of DM1, contributing to the prevention and minimization of long-term consequences of DM1, is in harmony. It is imperative to invest in the promotion of self-management of DM1 by adolescents in an early and effective way, contributing to their well-being and independence and self-management of this complex chronic disease.

## Figures and Tables

**Table 1:** Data extracted with the review.

Article (Authors/Year/Title/Evidence Level)	Population	Aims	Results
**Ksir, Wood, Hasni** **Sahli, Quinn and** **Ghardallou (2022)** [6] **Motivational interviewing to imrpove self-management in youth with type 1 diabetes: a randomized clinical trial** **Evidence level** **1.a. Systematic review of** **controlled trials** **randomized**	*n* = 66 teenagers between 13 and 18 years old with DM	To evaluate the effectiveness of a intervention of health (motivational interview), led by nurses, in order to will improve self-management of DM1 on the part in teenagers	Nurses taught, for six months, adolescents with DM1 and this had beneficial effects in reducing the consequences of DM1, as well as causing a substantial improvement in skills among adolescents, related to self-management of this chronic disease.
**Brito, Cunha, Silva** **and Queirós (2021)** [14] **Motivational interviewing and self-management among adolescentes with type 1 diabetes: an integrative review** **Evidence level** **4.d. Case study**	*n* = 10 studies	To analyze evidence scientifica bout the realization of interviews motivational and your influencein self-managementof the care of adolescents with DM1	The results showed positive impacts, following the motivational interviews, for self-management among adolescents with DM1, especially in relation to glycemic control and/or HbA1c levels.
**Beak and Kim (2021)** [15] **Factors Included in T1 DM Continuing Education for Korean School Nurses: A Systematic Review** **Evidence Level** **2.a. Systematic review of quasi-experimental studies**	*n* = 12 studies	To identify the key factors for inclusion in the continuous education of nurses on school health (in Korea) to improve DM 1 management by students	This study enabled us to understand the perspective of the health school nurse as a leader, demonstrating that they must be aware of laws requiring policies and protocols for the care for students with diabetes at school. Some necessary skills were identified to develop teaching in a school environment: the strengthening of these adolescents’ self-management competence, and facilitating networking with experts and peers. The perspective of the school health nurse as a leader, using a standardized evidence-based care approach specific to the treatment of T1 DM; self-management support, promotion of healthy habits in students and communication and collaboration between key stakeholders. At the time of the study, school teaching on T1 DM treatment was facing challenges due to COVID-19 and the associated need to change education methods. In this context, some treatments were carrid out through online platforms or e-learning adapted to therapeutic education, which can overcome some gaps.
**Hung, Huang, Lo and Cheng (2020)** [16] **The Self-Management** **Experiences of Adolescents with Type 1 Diabetes: A Descriptive Phenomenology Study** **Evidence level** **4.d. Case study**	*n* = 18 teenagers aged between 13 and 18 years old with DM1	To understand the experiences of self-management in teenagers with DM1 in Taiwan	This study found that self-management of DM1 by adolescents is influenced by parental behavior. Dietary diversity as well as stress contribute to these adolescents becoming incapable of quantifying carbohydrates, as well as adequate insulin units. In addition, overprotectveness among parents contributes to adolescents presenting an inner struggle about their independence. In this sense, the role of health professionals can be crucial in the development of therapeutic nursing education interventions in order to promote DM1 self-management among adolescents.
**Banca, Brandão, Sparapani, Souza, Neves, Cavicchioli, Lima and Nascimento (2020)** [17] **A Fun Way to Learn About Diabetes: Using Therapeutic Play in a Brazilian Camp** **Evidence level** **1.b. Systematic review of ERC and other study drawings**	*n* = 20 teenagers aged between 9 and 17 years old with DM1	To understand the benefits of Instructional Therapeutic Toy (ITT) group sessions performed by nurses in a Brazilian summer camp for young people’s understanding of the pathophysiology of DM1	Therapeutic game sessions for teenagers in a summer camp: drawings on insulin production; glycemic surveillance training in diabetes management; therapeutic play (TP) session as a safe space to share challenges with nurses and peers; and unraveling diabetes myths with the BT session improved the knowledge of young people and dispelled myths about the pathophysiology of DM1. While this teaching strategy was implemented in the diabetes camp, these sessions can be implemented and developed by nurses in other settings to provide age-appropriate diabetes education to pediatric patients. By educating young people with diabetes using age-appropriate strategies, nurses help them develop new skills and participate in their health care process.
**Evcimen, Uncu and Esen** **(2018)** [18] **Investigation of the Effect of Motivational Interviewing on Self Efficacy Levels in Adolescents with Type 1 Diabetes Mellitus** **Evidence level** **1.b. Systematic review of ERC and other study designs**	*n* = 66 teenagers aged between 11 and 18 years old with DM1	To investigate the effect of interviews in education for self-management of DM on self-efficacy, development of behaviors of styles of a healthy life and HbA1c in teenagers with DM1	This study showed that, when implementing educational interviews for DM1 self-management, there was a substantial increase in the total score of the self-efficacy scale based on the motivational interview and in the total score of the scale for developing healthy lifestyle behaviors, in comparison with the period prior to the interview. In summary, it was concluded that interviews based on DM1 self-management education increased self-efficacy in adolescents with DM1, favorably promoted healthy lifestyle behaviors and decreased hemoglobin HbA1c.

## Data Availability

Not applicable.

## Appendix A

**Table A1:** Ordered descriptors and respective Boolean operators for the search on the PubMed platform (with access to the Medline specialized database).

Search	Query	
**#1**	“Therapeutic education” [Title/Abstract] OR Education [Title/Abstract] OR Education [Term MesH]	266,956
**#2**	Nurs* [Title/Abstract] OR Nursing [Term MesH]	115,138
**#3**	“Self-management*” [Term MesH] MesH] OR “Self Management” [Title/Abstract] OR “Management Self” [Title/Abstract]	11,709
**#4**	Child* [Title/Abstract] OR Adolescen* [Title/Abstract] OR Teen* [Title/Abstract] OR Youth [Title/Abstract] OR “Young people” [Title/Abstract] OR Paediatric* [Title/Abstract] OR Pediatric* [Title/Abstract] OR Scholar [Title/Abstract] OR “Adolescent” [Term MesH] OR “Child” [Term MesH]	707,772
**#5**	“Type 1 Diabetes Mellitus” [Term MesH] OR “Type 1 Diabetes” [Title/Abstract] OR “Diabetes Mellitus Type 1” [Term MesH] OR Diabetes Type 1 [Title/Abstract]	19,041
**#6**	#1 AND #2 AND #3 AND #4 AND #5 AND	47
	Limited to language (English, Portuguese, Spanish)	37

MEDLINE (PubMed) survey conducted on 19 December 2022.
